# Influences of dynamic and static retrieval cues on memory for events

**DOI:** 10.3389/fcogn.2026.1714095

**Published:** 2026-03-17

**Authors:** Megan S. Smithwick, Alan W. Kersten, Kevin P. Darby, Julie L. Earles

**Affiliations:** 1 Department of Psychology, Florida Atlantic University, Boca Raton, FL, United States; 2 Wilkes Honors College, Florida Atlantic University, Jupiter, FL, United States

**Keywords:** associative memory, dynamic retrieval cues, event cognition, item memory, memory retrieval, recognition

## Abstract

Prior research on recognition memory has commonly used static cues (e.g., images) to evaluate familiarity-based item memory and recollection-based associative memory. Dynamic cues (e.g., videos) offer spatiotemporal information that may alter retrieval processes. In the present study, we examined how retrieval cue type (images vs. videos) and presentation duration affect memory accuracy and reaction times (RTs). Participants (*N* = 188) encoded video clips of actors performing actions. At retrieval, they viewed images or videos for a short (733 ms) or long (1,466 ms) duration. Test items included old (intact actor-action pairings) and conjunction (recombined actor-action pairings) items to evaluate associative memory, and new actor (unfamiliar actor, familiar action) and new action (familiar actor, unfamiliar action) items to assess item memory. Analyses of memory performance revealed that videos at retrieval facilitated the rejection of new action items, while simultaneously promoting a greater tendency to endorse new actor items compared to images. A hierarchical ex-Gaussian model indicated that short viewing durations at retrieval led to slower average RTs and increased the frequency of very long RTs, whereas static image cues were associated with greater RT variability and increased the prevalence of prolonged memory searches. Longer viewing durations reduced the occurrence of extended memory searches for associative decisions, particularly for conjunction items. Dynamic and static retrieval cues thus differentially influenced the familiarity of individual features of an event, whereas the associative binding of those features was primarily influenced by stimulus duration, regardless of whether those stimuli were static or dynamic.

## Introduction

As Gibson (1975) observed, “events are perceivable but time is not.” In everyday life, we often rely on events and the associated changes (e.g., motion, sound) to perceive the passage of time. Motion and the progression of change serve as indices of temporal passage and a gateway through which time becomes apparent. Static stimuli inherently lack these temporally dependent features that define dynamic stimuli, and these fundamental differences likely influence perception and other cognitive processes. For instance, stimuli containing motion are perceived as longer in duration than static stimuli, and this effect does not seem to be modulated by the amount of motion (Brown, 1995; Kaneko and Murakami, 2009; Matthews and Meck, 2016). Dynamic stimuli are perceived as more vivid

and attentionally engaging (Goldstein et al., 1982; Matthews and Meck, 2016) and can even decrease reaction times (RTs) for specific tasks, including emotion classification (Goldberg et al., 2015).

The spatiotemporal advantages of dynamic stimuli may also shape memory retrieval. Goldstein et al. (1982) conducted one of the first studies to evaluate static and dynamic retrieval cues and found that both stimulus types provided similar hits to old items; however, videos improved rejection of lures compared to images. This motion-driven improvement is often called the dynamic superiority effect, which occurs spontaneously, even persisting when the quantity of information is controlled for (e.g., using multiple static frames; Buratto et al., 2009; Matthews et al., 2007). Therefore, the benefit of dynamic stimuli is not based on the amount of information provided, but rather the types of information conveyed, including temporal and spatial cues. Indeed, dynamic stimuli seem to improve memory performance even under very brief presentation durations (e.g., 200–400 ms; Candan et al., 2015). Matthews et al. (2010) evaluated how static and dynamic cues influence memory accuracy of event details (i.e., people, actions, and backgrounds). Videos increased hits for old items, but also led to more false alarms to lures containing similar features to the original event, such as actors and backgrounds. Taken together, prior studies show mixed results regarding which kinds of event information benefit most from dynamic cues, and the extent to which static and dynamic stimuli may influence the binding of different elements in memory remains unclear.

Thus far, studies evaluating differences between static and dynamic cues have largely used stimuli extracted from Hollywood-style movies, making it difficult to cleanly separate the effects of these stimulus types on the two key processes supporting recognition memory: item and associative memory. Item memory is the ability to remember individual units of information (e.g., individual actors or actions). In contrast, associative memory is the ability to remember associations between items or units of information (e.g., actor-action pairings). The dual-process theories of memory propose that recognition involves two distinct processes with dissociable components and time courses. Item memory is thought to be supported by familiarity, a fast, automatic sense that something was previously encountered without recalling contextual information, such as spatial, temporal, or sensory details (Hockley and Consoli, 1999; Yonelinas, 2002). In contrast, associative memory typically requires recollection, a slower, more controlled process that involves retrieving contextual details. Although dual-process models differ in some respects, most assume these processes are initiated in parallel and primarily operate independently at retrieval (Dobbins et al., 2000; Graf and Mandler, 1984; Jacoby, 1991; Mandler, 1980; Yonelinas and Jacoby, 1994). Significantly, these processes unfold on different timescales, with familiarity-based item information becoming available within ~350 ms of stimulus presentation, whereas recollection-based associative information appears around 600–700 ms (Gronlund and Ratcliff, 1989; McElree et al., 1999). Performance for item memory can continue to increase until ~650 ms, whereas associative memory performance plateaus around 1,400 ms. Studies evaluating this time course used static retrieval cues (i.e., written words), leaving it unclear whether viewing duration may differentially influence retrieval across cue types. Brief exposures may constrain the temporal unfolding of dynamic cue (e.g., videos) information, whereas longer exposures allow for additional cues, which could be especially beneficial for associative memory. Contrastingly, static cues are temporally invariant, meaning all the information necessary to make a memory decision is available once processed, so viewing duration may be less impactful.

Prior research studies evaluating factors related to the dual process theories of recognition memory have overwhelmingly relied on static cues, including visually presented word pairs (Buchler et al., 2008; Bader et al., 2010; Castel and Craik, 2003; Kamp et al., 2018), objects paired with scenes (Fidalgo et al., 2016; Hayes et al., 2007; Luck et al., 2014), or faces paired with names (Guo et al., 2005; Naveh-Benjamin et al., 2004; Rentz et al., 2011; Sperling et al., 2003). In contrast, fewer studies have employed naturalistic, dynamic materials, such as videos (Kersten et al., 2008, 2018; Kersten and Earles, 2010). Thus, it remains unknown how static and dynamic cues might differentially influence item and associative memory. One possibility is that motion, or the lack thereof, may provide different types of information or direct attention to certain components of naturalistic events, thereby shaping which types of information are most readily retrieved. For example, in static displays, faces reliably capture and hold attention longer than other non-face objects (Bindemann et al., 2005; Theeuwes and Van der Stigchel, 2006), which may lead individuals to prioritize memory for facial identity over other event features, like an action being performed. Conversely, dynamic cues at retrieval may draw attention to body movements, such as actions being performed, and provide additional spatiotemporal cues that could even promote more accurate memory retrieval of actor-action bindings.

No studies have directly evaluated whether static and dynamic cues differentially influence accuracy and the temporal dynamics of item and associative recognition. To evaluate this, participants in the present study encoded naturalistic videos of actors performing actions. At retrieval, we manipulated cue type (image vs. video) and viewing duration (short: 733 ms vs. long: 1,466 ms) to examine effects on recognition accuracy and RTs. We tested item memory using new actor items (novel actor performing a familiar action) and new action items (familiar actor performing a novel action). Accuracy for the new actor and action items requires identifying whether individual components (i.e., the actor or action) were seen previously, rather than recalling a specific actor-action pairing. Associative memory was assessed with old items (same actor-action pairings) and conjunction items (novel recombination of familiar actors and actions). Distinguishing old and conjunction items thus requires participants to remember specific actor-action pairings.

RTs were analyzed with a hierarchical exponentially modified Gaussian model to evaluate the full RT distributions. This model decomposes the RT distribution into Gaussian (normal) and exponential distributions using three parameters (Hockley, 2008). These parameters include μ (mu), which represents the mean of the Gaussian distribution; σ (sigma), which is symmetrical and measures variability or spread around μ*;* and τ (tau), the mean of the exponential component, which is not symmetrical and captures the degree of positive skew. In the context of memory retrieval, lower μ and σ values indicate faster typical responses and more consistent processing of a retrieval cue and could suggest greater retrieval efficiency or automatic-like processing (Balota and Spieler, 1999). Lower τ values reflect less frequent trials with very long RTs, indicating a less effortful retrieval process or less response competition (Gholson and Hohle, 1968; Wixted and Rohrer, 1993; Wixted et al., 1997).

We expected videos to provide richer spatiotemporal information, facilitating better discrimination of old items from novel items (conjunction, new actor, and new action) compared to images. A comparison of performance with different types of novel items will provide evidence regarding the effects of spatiotemporal information on familiarity with the individual features of an event and a recollection of how those features were combined.

Regarding RTs, we predicted that associative memory items, particularly conjunction trials, would produce longer RTs than item memory trials (new actor and new action), reflecting additional time for associative memory retrieval. In the ex-Gaussian model, this should appear as higher μ and/or τ parameter values for associative items, especially for conjunction trials, compared to item memory trials. Finally, we anticipated videos would yield lower μ and/or τ than images, because motion may provide spatiotemporal cues that speed up and support less effortful retrieval.

## Method

### Participants

Participants were 188 undergraduate students (128 female, 58 male, and two non-responses; mean age = 20.77, SD = 3.67), receiving course credits or extra credit for participation. Participants were randomly assigned to one of four between-subjects conditions: short image (*n* = 47), long image (*n* = 47), short video (*n* = 46), and long video (*n* = 48). The Institutional Review Board of Florida Atlantic University approved the study, and all students provided written consent.

An *a priori* power analysis was conducted using G^*^Power 3.1 (Faul et al., 2007) to determine the required sample size. The analysis revealed that 188 participants were required to achieve 0.80 power to detect a significant within-between interaction between the within-subjects factor of item type (old vs. conjunction and new actor vs. new action) and the between-subjects factors (short image vs. long image vs. short video vs. long video). This estimate assumed a medium correlation among repeated measures (*r* = 0.25), a medium effect size (0.15), and an alpha level of 0.05.

### Materials

**Figure 1:**
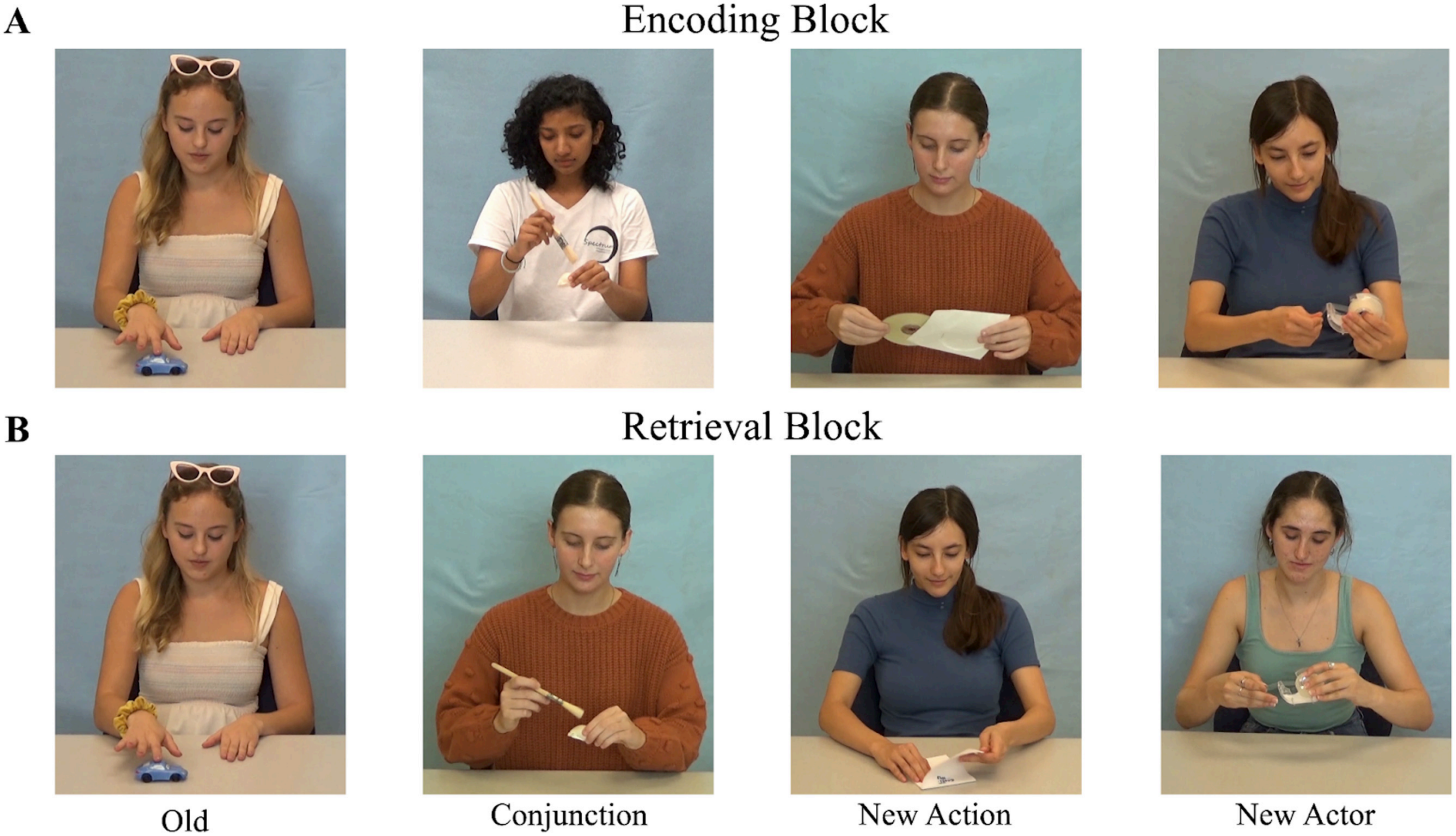
Images from example encoding **(A)** and retrieval **(B)** block items. The test items at retrieval included old (same actor and action pairing), conjunction (novel pairing of familiar actor and action), new action (familiar actor, new action), and new actor events (unfamiliar actor, familiar action).

The stimuli included videos of 64 female actors, each of whom performed two actions with objects (e.g., stretching a balloon, pinching a clothespin) for a total of 128 video clips that were 4–8 s (*M* = 5.42) in duration and 128 corresponding still frames taken from those videos. For each encoding video, we identified a single frame that clearly depicted the performed action; this still frame served as the image retrieval cue. For example, if the video at encoding depicted an actor stretching a balloon, the selected still frame showed the actor holding the balloon fully stretched. The short and long video stimuli were centered around the selected still frame to present an equal amount of time before and after that still frame. Each retrieval stimulus was presented for either 733 or 1,466 ms. These durations were based on prior study findings on the temporal availability of item and associative information (Gronlund and Ratcliff, 1989; McElree et al., 1999). Adobe Premiere Pro software was used to create the retrieval stimuli, using a frame rate of 30 frames per second. Because each frame has a duration of 33.33 milliseconds, durations had to be multiples of this value. Retrieval included four recognition item types (old, conjunction, new action, and new actor), each comprising 16 items to assess item and associative memory. To construct all four recognition item types, two actors were each filmed performing the same two actions. For example, actors A and B were filmed stretching a balloon and pinching a clothespin. Four different counterbalancing lists were used for the image and video conditions, such that across these lists, each actor and action was presented an equal number of times at encoding and retrieval, and served as each recognition item type once. Supplementary Figure 1 provides an illustration of the item type counterbalancing methodology. The old items consisted of the same actor-action pairings from encoding. The conjunction items recombined familiar actor-action pairings from the encoding block. New actor items presented the same action shown at encoding, but performed by an actor who was not studied at encoding. Lastly, new action times presented an actor shown at encoding performing a new action that was not shown at encoding. Figure 1 provides example images of the item types. Retrieval cue type and duration were manipulated between-subjects, whereas recognition test items were manipulated within-subjects.

### Procedure

SuperLab (6.5.1) was utilized to administer the experiment, comprising a 60-min session with a 25-min delay block between the encoding and retrieval blocks. After providing informed consent, participants were told their memory would be tested for the actors and the actions performed in each video. During encoding, participants viewed 48 videos of actors performing actions in a randomized order. Participants then completed a demographics questionnaire and filler tasks during a delay block.

In the retrieval block, participants were tested on their memory and were instructed that some videos or images contain the same actor-action pairings viewed at encoding, whereas others are different from what was seen at encoding. Participants were shown 64 images or videos of actors performing actions for 733 or 1,466 ms. After each cue, participants were asked, “Did you see this person perform this action in the first part of the experiment?” Participants were instructed to respond “yes” if they recalled seeing that same actor-action pairing previously and to respond “no” to any test items containing different actor-action pairings from what was shown previously or if they did not remember an actor or action. Participants made their responses by using designated buttons on a Cedrus response pad (RB−844) and were instructed to make their memory judgments as quickly and accurately as possible.

## Results

### Memory performance

A hierarchical logistic regression with Bayesian inference was implemented to model the probability of “yes” responses to the item types (old, conjunction, new actor, and new action), given the different cue types (image vs. video) and presentation durations (short vs. long). Fixed (group-level) effects were estimated for each variable with the intercept and all interactions. Random effects were estimated for the intercept of each participant to allow for individual variability. Bayesian inference was performed using Markov Chain Monte Carlo (MCMC) methods in the Numpyro library in Python (Phan and Pradhan, 2019), estimating the posterior distributions of coefficients and posterior-predictive contrasts (i.e., differences in predicted probabilities between conditions) with 95% highest density intervals (HDIs). The reported effects are posterior mean differences on the probability scale (*M*dif). Effects are considered credible when the 95% HDI excludes 0. The main text highlights the credible model effects for brevity. Refer to Supplementary Table 1 for the complete model estimates and Supplementary Figure 2 for a summary of the memory results across all conditions.

The logistic regression model revealed credible main effects of item type (old vs. lures), such that participants were more likely to provide “yes” responses to old items than to conjunction items [*M*dif = +0.371, 95% HDI (+0.337, +0.405)], new actor [*M*dif = +0.484, (+0.453, +0.516)], and new action items [*M*dif = +0.696, (+0.670, +0.721)]. Additionally, the probability of “yes” responses was higher to conjunction items compared to new actor items [conjunction vs. new actor; *M*dif = +0.113, (+0.076, +0.147)], conjunction compared to new action [conjunction vs. new action; *M*dif = +0.325, (+0.292, +0.360)], and for new actor compared to new action [new actor vs. new action; *M*dif = +0.212, (+0.183, +0.245)].

**Figure 2:**
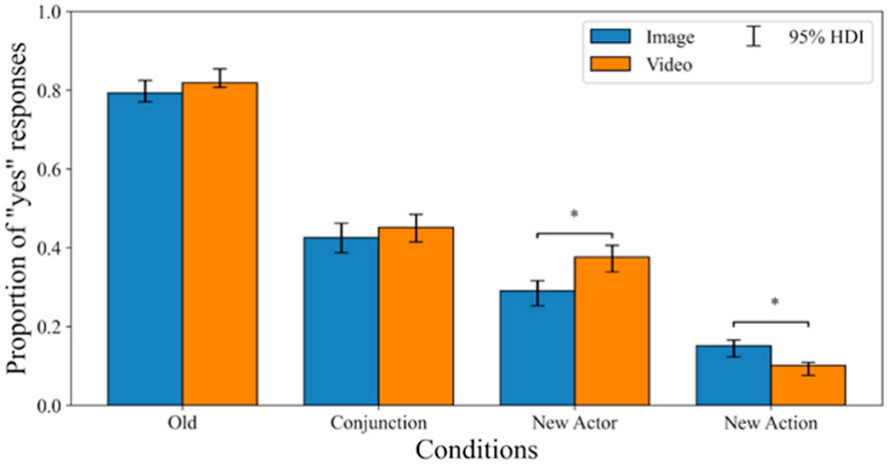
This figure displays the observed proportion of “yes” responses for each test item type shown at retrieval, separated by stimulus type (image vs. video). Whiskers show 95% HDIs, and asterisks indicate credible differences. Images facilitated the rejection of new actor items, whereas videos supported the rejection of new action items.

Regarding interaction effects, our primary research interests concern differences between associative memory (old vs. conjunction items) and item memory (new actor vs. new action items). Figure 2 displays the observed proportion of “yes” responses to the different retrieval cues and test item types, with model posterior 95% HDI whiskers, illustrating model fit to the observed data. There was a credible interaction between item type (new actor vs. new action) and cue type [image vs. video; *M*dif = −0.137, (−0.182, −0.093)]. Simple effects showed that within new actor items, videos led to a higher probability of “yes” responses than images [*M*dif = −0.087, (−0.132, −0.042)]. In contrast, images led to a higher probability of “yes” responses to new action items than videos [*M*dif = +0.051, (+0.024, +0.079)]. To evaluate whether this significant interaction could have been impacted by a change in response bias, we conducted a signal-detection criterion analysis comparing criterion placement (c) for cue type separately for old vs. new action items and old vs. new actor items. This analysis revealed that criterion placement for the old and new action items did not significantly differ between retrieval cue types, *t*(186) = −0.962, *p* = 0.3374. However, criterion placement differed significantly for the old vs. new actor comparison, *t*(186) = 3.018, *p* = 0.003, indicating that participants may have adopted a more liberal criterion for accepting new actor items as old when shown as videos (*M* = −0.325, SD = 0.403) compared to images (*M* = 0.150, SD = 0.385). Taken together, these findings suggest that videos supported better discrimination of old and new actions without evidence of a criterion shift, whereas the higher false alarm rate to new actor items in the video condition could reflect a criterion shift rather than improved discrimination for images.

### RT modeling

**Figure 3:**
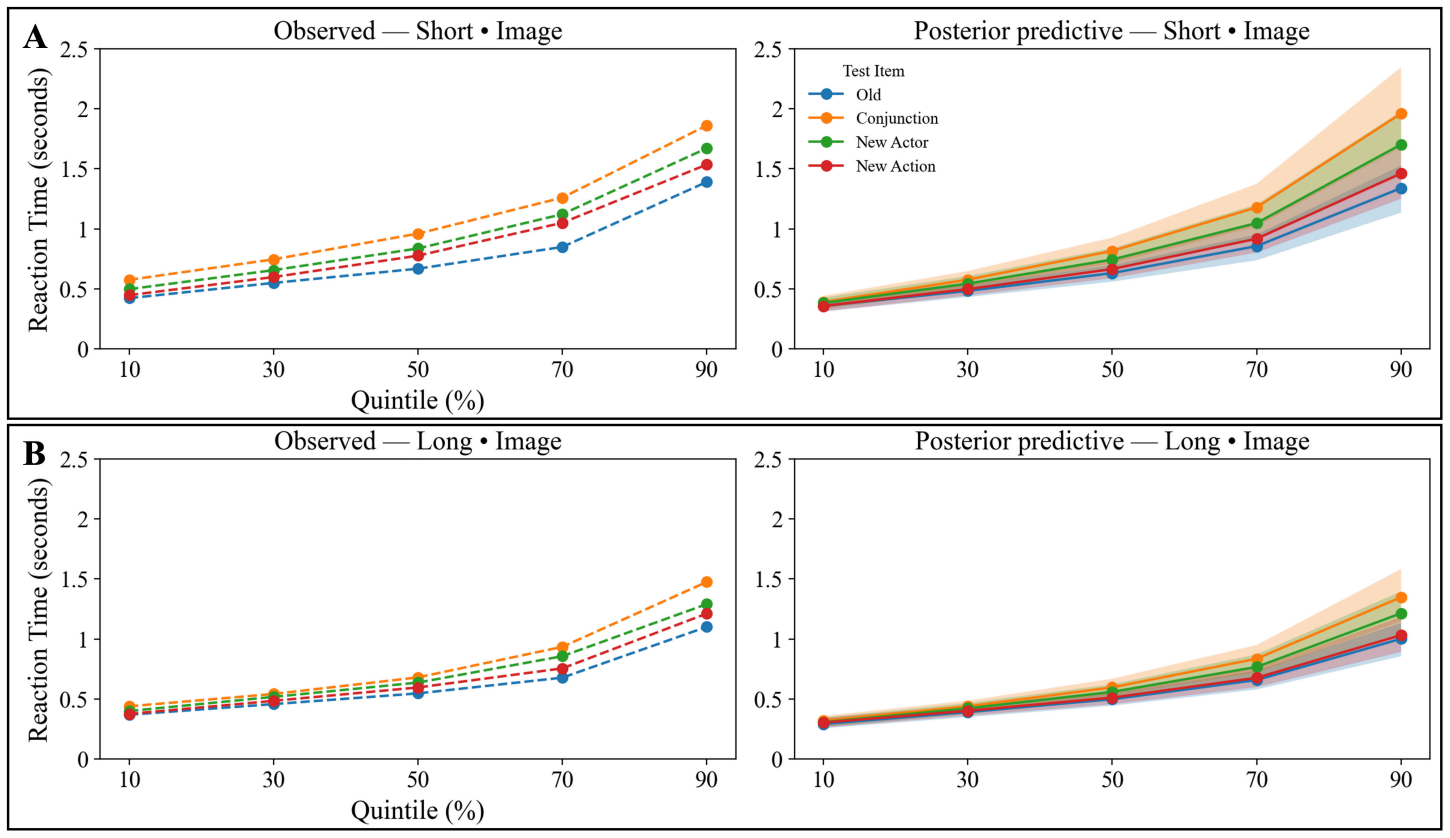
**(A, B)** are quintile plots for the short **(A)** and long **(B)** image conditions across test item types (old, conjunction, new actor, new action). The simulated data are derived from the posterior-predictive means from the hierarchical ex-Gaussian model, and the model prediction error bands represent 95% HDIs.

We implemented a hierarchical ex-Gaussian regression model to evaluate the full RT distribution. Bayesian inference was performed using MCMC methods in the Python Numpyro library to estimate the full posterior distribution for each participant. The model only included correct responses to the item types (e.g., “yes” to old, and “no” to conjunction, new actor, and new action). Subject-level estimations for μ, σ, and τ were made for each participant based on their RTs to each item type. Additional model information, including the full results, can be found in the Supplementary Materials. Figures 3 (images) and 4 (videos) present quintile plots (10th−90th percentiles) comparing observed and posterior-predictive RTs across duration, item, and cue types. The quintile plots show that old items generally yielded faster RTs (70th and 90th percentiles) than other item types, whereas conjunction and new actor items tended to be the slowest. The model supports these main effect patterns, revealing that old items (old vs. lures) led to a shorter exponential tail, indicating fewer trials with very long memory searches compared to conjunction [τ: *M*dif = −0.207 s, 95% HDI (−0.244 s, −0.168 s)], new actor [τ: = −0.133 s, (−0.164, −0.102)], and new action items [τ: *M*dif = −0.028 s, (−0.051, −0.003)]. In contrast, conjunction items led to more trials with very long memory searches compared to new actor items [conjunction vs. new actor; τ: *M*dif = +0.074 s, (+0.036, +0.113)] and new action items [conjunction vs. new action; τ: *M*dif = +0.180 s, (+0.141, +0.216)]. Lastly, new actors led to more trials with prolonged memory searches compared to new actions [new actor vs. new action; τ: *M*dif = 0.105 s, (+0.074, +0.137)]. Thus, item type differences were primarily reflected in the prevalence of prolonged retrieval processes, which also paralleled false alarm patterns: conjunction items exhibited the largest exponential tail, indicating a greater number of trials involving extended memory searches for correct rejections. Notably, conjunction items were also associated with higher false alarm rates than new actor and action items, suggesting that the increased retrieval demands not only produced more frequent, prolonged memory search trials on correct responses but also increased the likelihood of false alarms.

**Figure 4:**
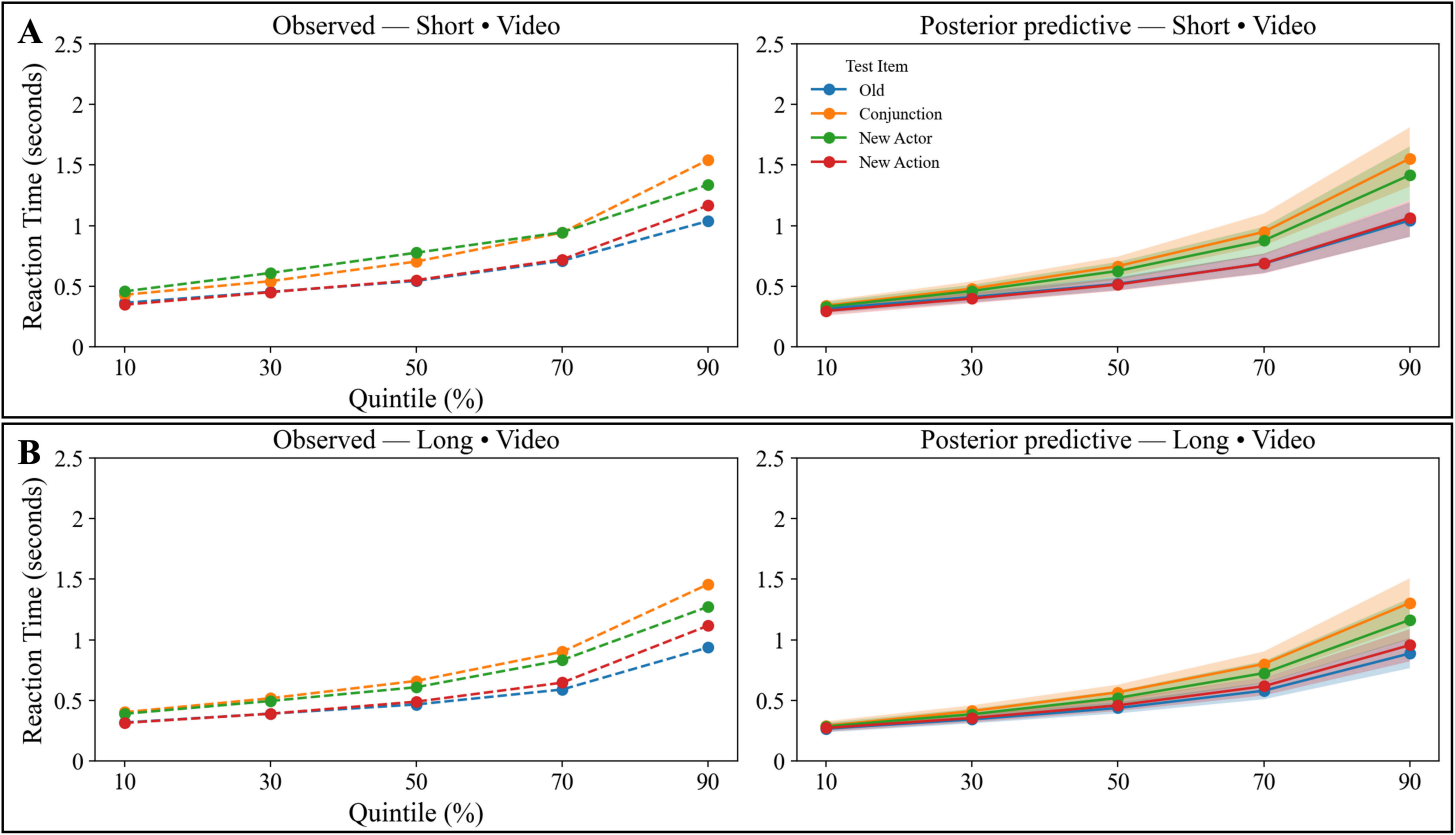
**(A, B)** are quintile plots for the short **(A)** and long **(B)** video conditions across test item types (old, conjunction, new actor, new action). The simulated data are derived from the posterior-predictive means from the hierarchical ex-Gaussian model, and the model prediction error bands represent 95% HDIs.

We expected a main effect of cue type, such that videos would facilitate faster RTs than images. In alignment with this prediction, the quintile plots show faster RTs for videos than images at the 70th and 90th percentiles. Furthermore, the model revealed that videos led to less variability in RTs than images [images vs. videos; σ: *M*dif = +0.021 s, (+0.005, +0.036)] and reduced the frequency of trials requiring prolonged memory searches [τ: *M*dif = +0.075 s, (+0.002, +0.156)]. Additionally, short viewing durations increased the average RT [short vs. long; μ: *M*dif = +0.043 s, (+0.009, +0.081)] and led to more trials with prolonged memory searches [τ: *M*dif = +0.124 s, (+0.051, +0.201)] compared to the long durations. Overall, longer viewing durations produced faster RTs with fewer trials requiring very long retrieval processes, whereas videos were associated with reduced variability in retrieval RTs and fewer trials with extended retrieval processes.

We also evaluated potential interaction effects related to associative memory (old vs. conjunction) and item memory (new actor vs. new action) A credible three-way interaction between associative memory item type, duration, and stimulus type emerged for RT variability [σ: Mdif = −0.055 s, 95% HDI (−0.097, −0.020)]. Simple effects showed that, at long durations, old items shown as images led to more variability compared to videos [σ: *M*dif = +0.021 s, (+0.003, +0.037)]. Contrastingly, conjunction items increased variability in RTs when viewed as long videos [σ: *M*dif = −0.018 s, (−0.035, −0.003)] compared to long images. This pattern of results was unexpected, and their interpretation is not straightforward. Additionally, a credible interaction between associative items and duration was found for very long RTs [τ: *M*dif = −0.084 s, (−0.163, −0.009)]. Simple effects revealed that longer viewing durations reduced the prevalence of extended memory search trials for old items [short vs. long; τ: *M*dif = +0.086 s, (+0.024, +0.148)] and conjunction items [τ: *M*dif = +0.170 s, (+0.059, +0.279)], such that long durations disproportionately reduced the occurrence of trials with extended searches for conjunction items. Overall, these findings indicate that increasing viewing duration for associative memory items reduces the frequency of trials involving prolonged, effortful retrieval, particularly for conjunction items.

## Discussion

The present study found that retrieval cue type (static images vs. dynamic videos) and viewing duration (short: 733 ms vs. long: 1,466 ms) influenced recognition accuracy and the temporal dynamics of memory retrieval. The results of this study revealed distinct patterns of effects of retrieval cue type on memory accuracy. Specifically, familiarity-related differences were shown in participants' ability to reject lures containing novel features (i.e., new actor and action). Thus, dynamic and static cues appeared to differentially influence memory for individual event features, rather than the associative binding of those features. In contrast, viewing durations primarily influenced associative retrieval by reducing the frequency of trials involving prolonged memory search processes, particularly for conjunction items.

Effects of retrieval cue type on familiarity were evident. For new actor lures, participants adopted a more liberal response criterion when shown as videos compared to images, indicating that dynamic cues increased the tendency to endorse new actor lures containing familiar actions as old. In contrast, videos improved the rejection of new action items, compared to images. A dynamic advantage for recognition of facial identity (O'Toole et al., 2002; Richoz et al., 2024) or expressions (Ambadar et al., 2005; Dobs et al., 2018) primarily emerges under degraded viewing conditions, whereas under typical viewing conditions, static cues can outperform dynamic ones, consistent with the present results. These findings may also reflect how motion can direct attention to different aspects of an event, ultimately influencing retrieval accuracy. For instance, the lack of motion in the images may have directed attention to the most salient aspect of the image, the actor's identity, consistent with prior findings that faces capture and engage attention for longer than other non-face objects (Bindemann et al., 2005; Theeuwes and Van der Stigchel, 2006). This could explain participants' improved rejection of new actor items when shown images. Conversely, the action-related motion in the video cues may have captured participants' attention and directed attention toward action information, improving rejection of new action lures while also promoting a liberal bias to endorse familiar actions performed by new actors.

Prior research has demonstrated study-test congruency effects, such that recognition performance is best when stimulus format at encoding and retrieval is congruent rather than incongruent (Buratto et al., 2009). Discrimination between studied items and lures is enhanced when dynamic stimuli are presented at both study and test, compared with single or even multiple static images. In the present study, however, presenting videos at both encoding and retrieval did not produce a general improvement in memory performance relative to videos at encoding and images at retrieval. One possibility is that study-test incongruency effects were present in both retrieval conditions due to the stimulus construction. The retrieval stimuli consisted of portions of video or still frames taken from the encoding videos, often drawn from the middle portions of the original event and were presented for shorter durations than during encoding. Thus, neither the image nor the video retrieval stimuli constituted a fully congruent repetition of the original encoding experience.

Our results differ from those of Matthews et al. (2010), who found higher false alarms for lures featuring familiar actors with new actions and/or backgrounds when presented as videos rather than images. In contrast, in the present study, we found that participants were better at rejecting items involving new actions when they were presented as videos rather than images. This discrepancy likely reflects differences in study design and/or test item type construction. Matthews et al. used the same stimulus (e.g., images) at encoding and retrieval, and lures exhibiting varying levels of similarity (e.g., familiar actors with new actions and/or backgrounds) to the studied item. Conceptually, Matthews et al. evaluated which stimulus (image vs. video) yielded better memory accuracy. In contrast, we examined the influences of cue type on supporting the retrieval of a dynamic event.

Retrieval cue type did not differentially influence item and associative memory RTs. We had predicted that associative trials would yield slower RTs than item memory trials and that long durations would improve discrimination of associative memory items, as it takes longer to become available (Gronlund and Ratcliff, 1989; McElree et al., 1999); however, RT results did not reflect these predictions. Instead, RTs mirrored response accuracy. Old items showed the fewest trials involving very prolonged memory searches, consistent with relatively efficient retrieval. In contrast, conjunction lures produced both the highest false alarm rates and a greater prevalence of trials requiring extended retrieval for correct memory judgments compared to all item types, suggesting that conjunction discrimination imposes especially high retrieval demands and possibly reflects increased response competition. Notably, longer viewing durations reduced the occurrence of extended memory searches for associative memory items, with a larger reduction for conjunction lures, suggesting that additional temporal support was particularly beneficial under higher retrieval demands and increased response competition, but this effect did not vary depending on whether retrieval cues were static images or dynamic videos.

It is possible that differences in memory performance for images and videos were attenuated through mnemonics, like mental simulation. When presented with an image depicting the central part of an action, kinematic mental simulation can occur, meaning the action's spatiotemporal trajectory is simulated, supporting action recognition judgments when motion is absent (Ianì et al., 2023). Related to this, the function gaze reinstatement theory suggests that eye movements at retrieval can even reinstate spatiotemporal context available at encoding (Kragel and Voss, 2021; Wynn et al., 2019). Importantly, the simulation of actions is improved when image cues are provided compared to written words, consistent with the idea that cues providing a closer approximation to the naturalistic event afford richer and superior reconstruction (Ianì et al., 2019). In support of this, the present study found that dynamic cues, which are more ecologically valid than static images, yielded less variable RTs and decreased the frequency of very long memory searches for correct decisions.

Although we encouraged participants to make their memory judgments quickly, we also provided an unlimited response window. RT analyses showed that short viewing durations were slower on average and resulted in more frequent extended searches to provide correct judgments. This finding may reflect that, without a time constraint, participants may have compensated for reduced cue exposure by engaging in a more effortful memory search or utilizing mnemonic strategies to achieve accuracy similar to the long duration condition. Instituting response deadlines could lead to additional downstream effects on accuracy for short durations and cue types.

Several considerations also motivate directions for future research. First, although participants were encouraged to respond quickly, the response window was unlimited, which may have allowed for compensatory strategies to offset reduced cue exposure in the image and short viewing duration conditions. The implementation of response deadlines could more directly assess how retrieval cue type and viewing duration influence accuracy and decision dynamics. Second, incorporating confidence ratings would provide a more systematic means of dissociating familiarity-based responses from recollection-based responses. Confidence ratings were not collected in the present study in order to maintain a streamlined design focused on obtaining precise RTs for recognition decisions. Prior work has shown that requiring retroactive confidence ratings can significantly increase decision time (Baranski and Petrusic, 2001; Petrusic and Baranski, 2003).

## Conclusion

We found that static (images) and dynamic (videos) retrieval cues, along with viewing duration (short: 733 ms; long: 1,466 ms), shaped both what was remembered and the time course of memory decisions. Dynamic cues supported better discrimination of new action items, whereas for new actor items, they promoted a liberal tendency to endorse as old, and images supported more conservative responses. Ex-Gaussian modeling of RT distributions showed that longer viewing durations produced faster RTs and reduced the prevalence of prolonged memory search trials, particularly for conjunction items. Videos at retrieval were associated with reduced RT variability and less frequent extended memory searches. Together, these findings suggest that dynamic and static cues primarily influence the recognition of individual event features, whereas the associative binding of those features is primarily a function of stimulus duration, regardless of whether stimuli are static or dynamic.

## Data Availability

The raw data supporting the conclusions of this article will be made available by the authors, without undue reservation.
