# Supplementary material for: Influences of dynamic and static retrieval cues on memory for events
Source: Front Cognit. 2026 Mar 17;5:1714095. doi: 10.3389/fcogn.2026.1714095 (PMC13281046; doi:10.3389/fcogn.2026.1714095)
Supplement: Supplementary file 1 [file Data_Sheet_1.docx]

**Supplemental.**

Below is an equation depicting the probability density function of the ex-Gaussian distribution:

$$f (x | \mu,\sigma,\tau)= \frac{1}{\tau} exp\left\{ \frac{\mu- x}{\tau}+ \frac{\sigma^{2}}{{2\tau}^{2}} \right\} \Phi\left( \frac{x - \mu}{\sigma}- \frac{\sigma}{\tau} \right)$$

In this equation, $x$ represents the observed reaction time and $\Phi$ denotes the cumulative distribution function (CDF) of the standard normal distribution, transforming a normal variable into a probability.

Priors for Bayesian regression models:

We modeled the probability of “Yes” responses with a binomial likelihood, $y_{i}\sim Bernoulli({logit}^{-1}(\eta_{i}))$, where $\eta_{i}=x_{i}^{\top}\beta+b_{s}$. Here, $x_{i}$ is the design matrix containing 16 fixed effect regressors, including an intercept, item-type dummies and their interactions with duration and stimulus type, along with main effects of duration and stimulus type and their interaction. These 16 regressors each used a standard normal prior: $\beta_{k}\sim Normal(0,1)$. We also included random intercepts for each subject: $b_{s}\sim Normal(0, HalfNormal(1))$. These subject intercepts were implemented with a non-centered parameterization. Similarly, we modeled reaction times with an ex-Gaussian likelihood, $y_{i}\sim ExGaussian(\mu_{i},\sigma_{i},\tau_{i})$. Each of the parameters $\mu_{i}$, $\sigma_{i}$and $\tau_{i}$were modeled as a regression of form $log(\eta_{i})=x_{i}^{\top}\beta+b_{s}$, where $\eta\in\{\mu, \sigma, \tau\}$. $x_{i}$ is the design matrix containing the same 16 regressors as above, with the same priors for $\beta$ and $b_{s}$ as for the binomial model described above.


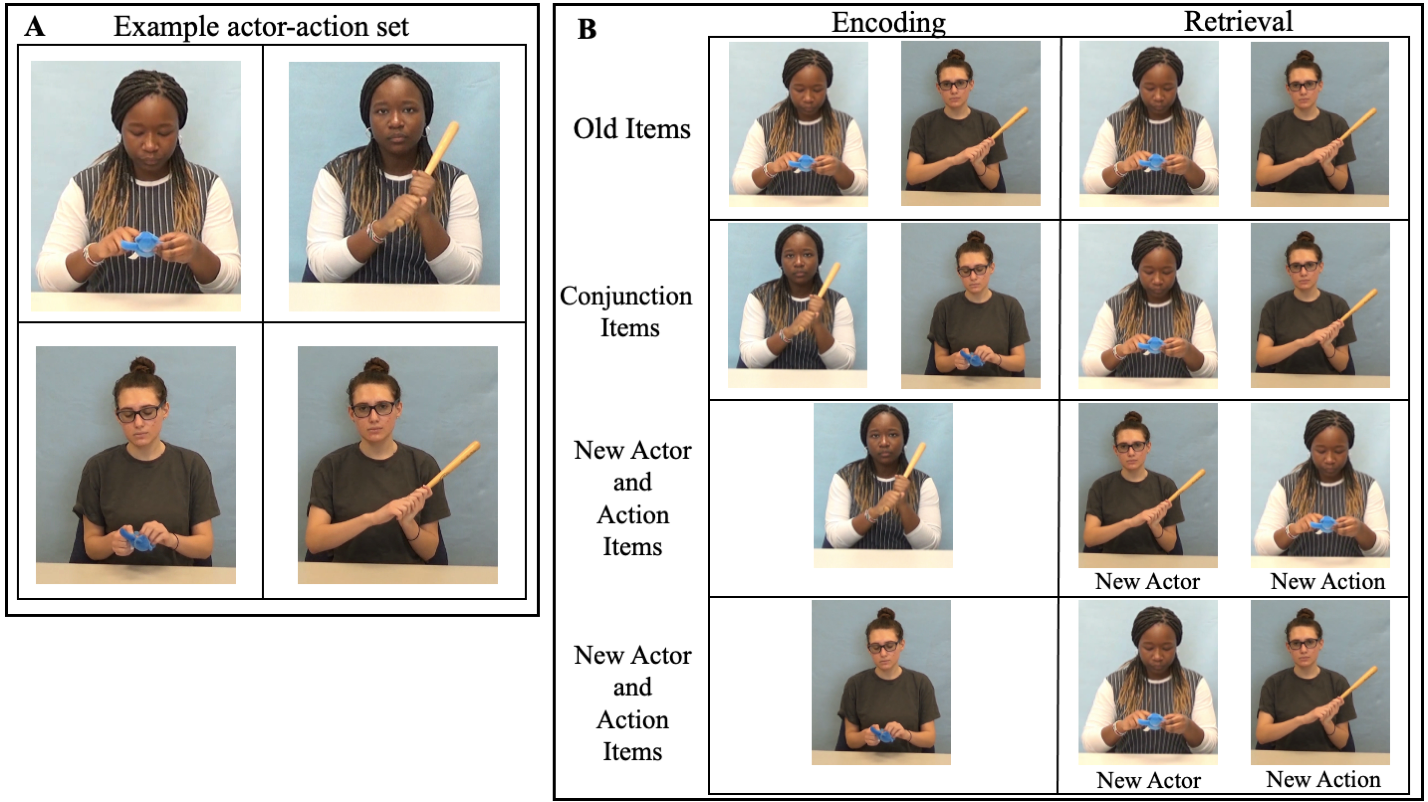


*Supplemental Figure 1.*(A) Example of an actor-action set used to create the four recognition item types. Two actors were filmed performing the same two actions, allowing each actor and action to be presented as old, conjunction, new actor, and new action items. (B) Example of the four between-subjects counterbalancing condition lists. Across encoding and retrieval, each actor and action is presented an equal number of times, and serves as an old, conjunction, new actor, and new action item a single time across the full counterbalanced design.


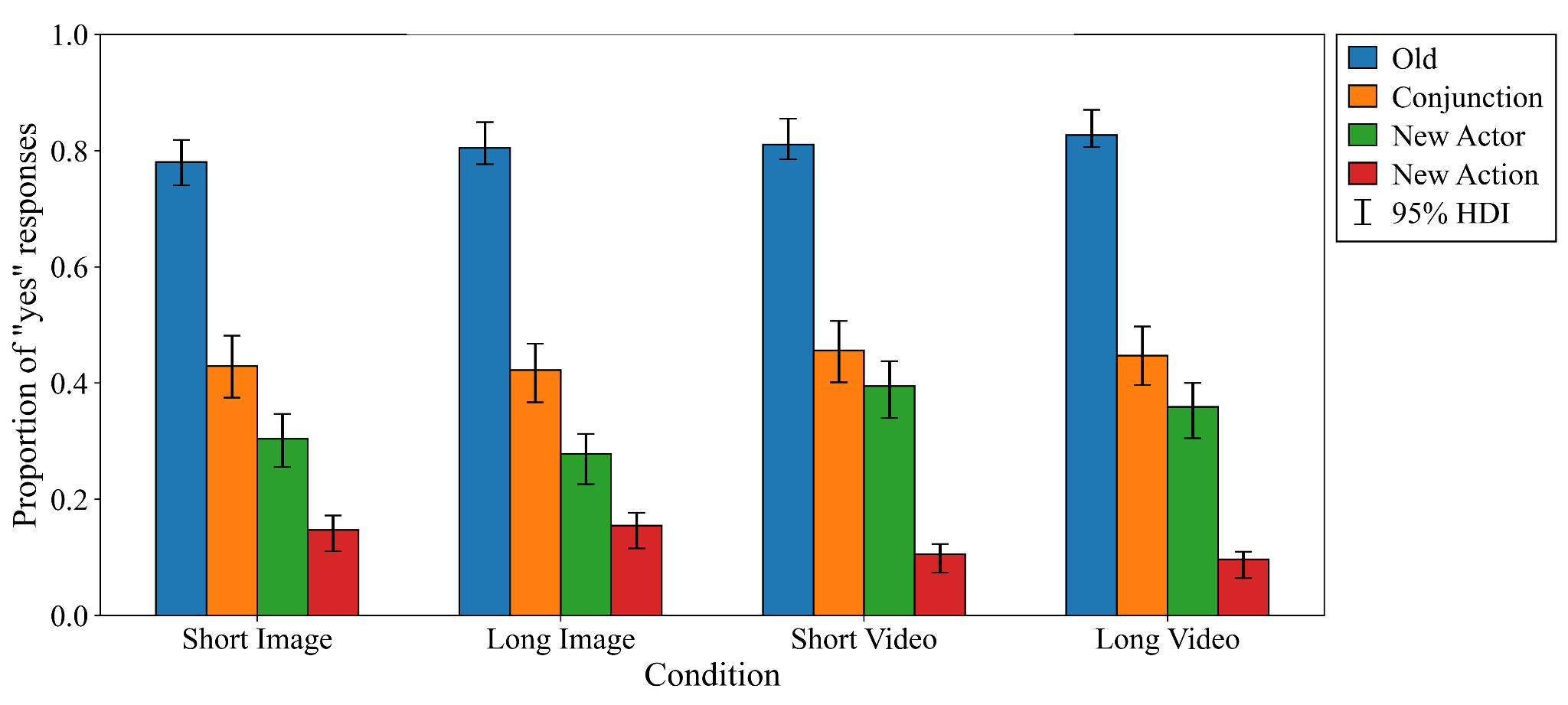


*Supplemental Figure 2.* This figure displays the observed proportion of “yes” responses for each test item type shown at retrieval, separated by duration (short: 733 ms vs. long: 1,466 ms) and Cue type (image vs. video). Whiskers show 95% HDIs. Regardless of duration, images facilitated the rejection of new actor items, whereas videos supported the rejection of new action items.

Supplemental Table 1

*Regression Modeling Predicting “yes” Responses*

| Comparison | Mean Difference | HDI Lower | HDI Upper | Credibility |
| --- | --- | --- | --- | --- |
| Old vs. Conjunction | + 0.371 | + 0.337 | + 0.405 | Credible |
| Old vs. New Actor | + 0.484 | + 0.453 | + 0.516 | Credible |
| Old vs. New Action | + 0.696 | + 0.670 | + 0.721 | Credible |
| Conjunction vs. New Actor | + 0.113 | +0.076 | + 0.147 | Credible |
| Conjunction vs. New Action | + 0.325 | +0.292 | + 0.360 | Credible |
| New Actor vs. New Action | + 0.212 | +0.183 | + 0.245 | Credible |
| Long vs. Short Duration | - 0.006 | - 0.035 | + 0.026 | Non-Credible |
| Video vs. Image Cue | + 0.024 | - 0.005 | + 0.054 | Non-Credible |
| Duration (Short – Long) x Cue (Image – Video) | + 0.008 | - 0.052 | + 0.067 | Non-Credible |
| Item (Old - Conjunction) x Duration (Short – Long) | - 0.036 | - 0.083 | + 0.013 | Non-Credible |
| Item Type (New Actor – New Action) x Duration (Short – Long) | + 0.031 | - 0.014 | + 0.076 | Non-Credible |
| Item Type (Old - Conjunction) x Cue (Image – Video) | - 0.006 | - 0.056 | + 0.041 | Non-Credible |
| Item Type (New Actor - New Action) x Cue (Image – Video) | - 0.137 | - 0.182 | - 0.093 | Credible |
| Item Type (New Actor) x Cue (Image – Video) | - 0.087 | - 0.132 | - 0.042 | Credible |
| Item Type (New Action) x Cue (Image – Video) | + 0.051 | + 0.024 | + 0.079 | Credible |
| Item Type (Old - Conjunction) x Cue (Image – Video) x Duration (Short – Long) | - 0.018 | - 0.107 | + 0.092 | Non-Credible |
| Item Type (New Actor - New Action) x Cue (Image – Video) x Duration (Short – Long) | + 0.009 | - 0.080 | + 0.096 | Non-Credible |

*Note.* Posterior model comparisons for the predicted “yes” responses to the different test items (old, conjunction, new actor, new action), retrieval cue types (image vs. video), and viewing durations (short vs. long).

Supplemental Table 2

*Ex-Gaussian Modeling of Reaction Times*

| Parameter Family | Comparison | Mean Difference | HDI Lower | HDI Upper | Credibility |
| --- | --- | --- | --- | --- | --- |
| *μ* | Old vs. Conjunction | + 0.002 s | - 0.010 s | + 0.013 s | Non-Credible |
| *μ* | Old vs. New Actor | + 0.000 s | - 0.009 s | + 0.012 s | Non-Credible |
| *μ* | Old vs. New Action | + 0.004 s | - 0.005 s | + 0.012 s | Non-Credible |
| *μ* | Conjunction vs. New actor | - 0.002 s | - 0.014 s | + 0.012 s | Non-Credible |
| *μ* | Conjunction vs. New Action | + 0.001 s | - 0.010 s | + 0.014 s | Non-Credible |
| *μ* | New Actor vs. New Action | + 0.003 s | - 0.007 s | + 0.014 s | Non-Credible |
| *μ* | Short vs. Long Duration | + 0.043 s | + 0.009 s | + 0.081 s | Credible |
| *μ* | Image vs. Video Cue | + 0.036 s | - 0.006 s | + 0.073 s | Non-Credible |
| *μ* | Duration (Short – Long) x Cue (Image – Video) | - 0.009 s | - 0.085 s | + 0.058 s | Non-Credible |
| *μ* | Item (Old - Conjunction) x Duration (Short – Long) | + 0.007 s | - 0.016 s | + 0.028 s | Non-Credible |
| *μ* | Item Type (New Actor – New Action) x Duration (Short – Long) | + 0.015 s | - 0.005 s | + 0.036 s | Non-Credible |
| *μ* | Item Type (Old - Conjunction) x Cue (Image – Video) | + 0.001 s | - 0.021 s | + 0.022 s | Non-Credible |
| *μ* | Item Type (New Actor - New Action) x Cue (Image – Video) | - 0.004 s | - 0.025 s | + 0.017 s | Non-Credible |
| *μ* | Item Type (Old - Conjunction) x Cue (Image – Video) x Duration (Short – Long) | - 0.014 s | - 0.055 s | + 0.031 s | Non-Credible |
| *μ* | Item Type (New Actor - New Action) x Cue (Image – Video) x Duration (Short – Long) | + 0.010 s | - 0.031 s | + 0.052 s | Non-Credible |
| *σ* | Old vs. Conjunction | + 0.004 s | - 0.007 s | + 0.015 s | Non-Credible |
| *σ* | Old vs. New Actor | + 0.003 s | - 0.007 s | + 0.014 s | Non-Credible |
| *σ* | Old vs. New Action | - 0.003 s | - 0.012 s | + 0.005 s | Non-Credible |
| *σ* | Conjunction vs. New Actor | - 0.000 s | - 0.013 s | + 0.012 s | Non-Credible |
| *σ* | Conjunction vs. New Action | - 0.007 s | - 0.018 s | + 0.005 s | Non-Credible |
| *σ* | New Actor vs. New Action | - 0.006 s | - 0.017 s | + 0.004 s | Non-Credible |
| *σ* | Short vs. Long Duration | + 0.014 s | - 0.002 s | + 0.030 s | Non-Credible |
| *σ* | Image vs. Video Cue | + 0.021 s | + 0.005 s | + 0.036 s | Credible |
| *σ* | Duration (Short – Long) x Cue (Image – Video) | + 0.001 s | - 0.029 s | + 0.031 s | Non-Credible |
| *σ* | Item (Old - Conjunction) x Duration (Short – Long) | + 0.004 s | - 0.017 s | + 0.026 s | Non-Credible |
| *σ* | Item Type (New Actor – New Action) x Duration (Short – Long) | + 0.002 s | - 0.019 s | + 0.023 s | Non-Credible |
| *σ* | Item Type (Old – Conjunction) x Cue (Image – Video) | + 0.011 s | - 0.010 s | + 0.031 s | Non-Credible |
| *σ* | Item Type (New Actor – New Action) x Cue (Image – Video) | + 0.012 s | - 0.008 s | + 0.033 s | Non-Credible |
| *σ* | Item Type (Old – Conjunction) x Cue (Image – Video) x Duration (Short – Long) | - 0.055 s | - 0.097 s | - 0.020 s | Credible |
| *σ* | Item Type (Old – Conjunction) x Cue (Image) x Duration (Long) | + 0.021 s | + 0.003 s | + 0.037 s | Credible |
| *σ* | Item Type (Old – Conjunction) x Cue (Video) x Duration (Long) | - 0.018 s | - 0.035 s | - 0.003 s | Credible |
| *σ* | Item Type (New Actor – New Action) x Cue (Image – Video) x Duration (Short – Long) | - 0.008 s | - 0.045 s | + 0.034 s | Non-Credible |
| 𝜏 | Old vs. Conjunction | - 0.207 s | - 0.244 s | - 0.168 s | Credible |
| 𝜏 | Old vs. New Actor | - 0.133 s | - 0.164 s | - 0.102 s | Credible |
| 𝜏 | Old vs. New Action | - 0.028 s | - 0.051 s | - 0.003 s | Credible |
| 𝜏 | Conjunction vs. New Actor | + 0.074 s | + 0.036 s | + 0.113 s | Credible |
| 𝜏 | Conjunction vs. New Action | + 0.180 s | + 0.141 s | + 0.216 s | Credible |
| 𝜏 | New Actor vs. New Action | + 0.105 s | + 0.074 s | + 0.137 s | Credible |
| 𝜏 | Short vs. Long Duration | + 0.124 s | + 0.051 s | + 0.201 s | Credible |
| 𝜏 | Image vs. Video Cue | + 0.075 s | + 0.002 s | + 0.156 s | Credible |
| 𝜏 | Duration (Short – Long) x Cue (Image – Video) | - 0.118 s | - 0.262 s | + 0.026 s | Non-Credible |
| 𝜏 | Item (Old - Conjunction) x Duration (Short – Long) | + 0.002 s | - 0.118 s | + 0.114 s | Non-Credible |
| 𝜏 | Item Type (New Actor – New Action) x Duration (Short – Long) | - 0.084 s | - 0.163 s | - 0.009 s | Credible |
| 𝜏 | Item Type (Old) x Duration (Short – Long) | + 0.086 | + 0.024 | + 0.148 | Credible |
| 𝜏 | Item Type (Conjunction) x Duration (Short – Long) | + 0.170 | + 0.059 | + 0.279 | Credible |
| 𝜏 | Item Type (Old - Conjunction) x Cue (Image – Video) | - 0.010 s | - 0.094 s | + 0.062 s | Non-Credible |
| 𝜏 | Item Type (New Actor - New Action) x Cue (Image – Video) | - 0.029 s | - 0.090 s | + 0.034 s | Non-Credible |
| 𝜏 | Item Type (Old - Conjunction) x Cue (Image – Video) x Duration (Short – Long) | - 0.071 s | - 0.225 s | + 0.073 s | Non-Credible |
| 𝜏 | Item Type (New Actor - New Action) x Cue (Image – Video) x Duration (Short – Long) | + 0.043 s | - 0.164 s | + 0.084 s | Non-Credible |

*Note.* Ex-Gaussian Posterior model parameter comparisons (*μ, σ,* 𝜏) for correct responses to the different test items (old, conjunction, new actor, new action), retrieval cue types (image vs. video), and viewing durations (short vs. long).
